# Endoscopic submucosal excavation for gastric muscularis propria tumours less than 10 mm in diameter: What are the risk factors responsible for perforation?

**DOI:** 10.1371/journal.pone.0319245

**Published:** 2025-02-28

**Authors:** Zhaohui Liu, Hualin Li, Jiwen Deng, Ruinuan Wu

**Affiliations:** 1 Department of Gastroenterology, The First Affiliated Hospital of Shenzhen University, Shenzhen Second People’s Hospital, Shenzhen, China; 2 Department of Medicine, Shenzhen University, Shenzhen, China; 3 Department of Pathology, The First Affiliated Hospital of Shenzhen University, Shenzhen Second People’s Hospital, Shenzhen, China; Acibadem Maslak Hospital: Acibadem Maslak Hastanesi, TÜRKIYE

## Abstract

**Objective:**

To explore the risk factors for perforation during endoscopic submucosal excavation (ESE) for gastric muscularis propria tumours less than 10 mm in diameter. This study provides clinical guidance for preventing the occurrence of intraoperative adverse events.

**Methods:**

Samples of gastric muscularis propria tumours less than 10 mm in diameter were removed via ESE at Shenzhen Second People’s Hospital and were collected from June 2023 to August 2024. The general clinical characteristics of the patients, location, size, growth pattern, and pathology of the tumours, operation time, resection time, perforation incidence and bleeding incidence were analysed, and logistic regression was used to calculate the risk factors for perforation and bleeding.

**Results:**

A total of 102 patients were included in this study. The tumours were successfully removed from all patients. The mean age was 52.28 ± 11.84 years. There were 34 (33.33%) males. The mean size was 6.96 ± 1.82 mm. 89 (87.25%) tumours exhibited an intraluminal growth pattern. In total, 79 (77.45%) tumours were in the gastric body, and 23 (22.55%) tumours were in the gastric fundus. The mean operation time was 35.26 ± 23.15 min, and the mean resection time was 27.88 ± 21.77 min. A total of 55 (53.92%) tumours were leiomyoma, and 43 (42.16%) tumours were GIST. There were 4 (3.92%) tumours classified as other lesions. 41 (40.20%) patients experienced intraoperative bleeding, all of which had minor bleeding and successful haemostasis under endoscopy.There were 27 (26.47%) concurrent perforation, of which,24(88.89%) were diagnosed as GIST, and 3(11.11%) were diagnosed as leiomyoma. All perforations were successfully managed with an endoscopic suture. According to the multivariate regression analysis, a pathologic diagnosis was a risk factor for perforation. When the pathological diagnosis is GIST, the risk of perforation increases (PE = 18.632, 95% CI 4.571 ~ 75.941; p < 0.001). Gender,age,tumor size, growth pattern, location, and resection time were not found to be risk factors for perforation.all of observed factors were not the risk factors for bleeding.

**Conclusion:**

ESE is an effective removal method for gastric muscularis propria tumours less than 10 mm in diameter.Intraoperative bleeding and perforation are common.However, these complications are controllable.GIST is an independent risk factor for the perforation.When the tumour is diagnosed as a GIST, the incidence of perforation is significantly increased.

## Introduction

There are many endoscopic methods for treating gastric muscularis propria tumours. Endoscopic submucosal excavation (ESE) is a commonly used resection method [1,2]. Bleeding and perforation during ESE are clinical problems that not only increase the difficulty of the operation and prolong operation time but also may lead to serious adverse events such as abdominal infection [3]. Evaluating the risk factors for perforation and bleeding during ESE has important clinical value for predicting and preventing intraoperative adverse events [4].

A increasing number of studies [5–8] indicate that resection of gastric muscularis propria tumours less than 10mm in diameter is warranted.Due to the small tumor size, this can lead to the increased difficulty of endoscopic operation.This is because after the incision of the mucosa and submucosa, it is often difficult to find the tumour body, and sometimes bleeding or perforation occurs when seek the tumour, which further aggravates the difficulty of the operation.The effect of endoscopic resection and risk factors of related complications of tumours less than 10mm is of positive significance for better clinical diagnosis and treatment.In this study, we retrospectively collected gastric muscularis propria tumours that were removed via ESE at the Second People’s Hospital. The risk factors for intraoperative perforation were evaluated via logistic regression. The results are reported below.

## Materials and methods

### Study design and ethics

This was a single-centre retrospective study conducted at Shenzhen Second People’s Hospital. The study was conducted in accordance with the 2008 revision of the Helsinki Declaration and was approved by the Ethics Committee of the Second People’s Hospital of Shenzhen.

The clinical data of patients who underwent ESE for the resection of gastric muscularis propria tumours less than 10 mm in diameter between June 2023 and August 2024 were collected, and informed consent was obtained from each patient prior to endoscopic resection. The requirement to obtain consent to use data obtained by the institution was waived due to the retrospective nature of the study.

### Indications for ESE

The operative indications are following: (1) gastric muscularis propria tumours; (2) tumours less than 10 mm in diameter in endoscopic ultrasound images; (3) patients with preoperative CT or MR images showing no lymph node or distant metastasis; and (4) patients who can tolerate endoscopic surgery. The operator decides whether to place ESE.

### Instruments

An endoscopic image processor (Olympus, Japan, CLV-290 sl), therapeutic gastroscope (Olympus, HQ260J,Japan), high-frequency electrical generator (Elbo,VIO300D, Germany), snare (Boston Science,M00561231,USA),haemostatic clip (Nanjing minimally invasive,China, POCC-D-26-195), and mucosal resection knife (Anre, China, AMH-TM-B0525) were used.

### ESE procedure

A transparent cap was installed at the front of the endoscope, and a tumour was found in the stomach. A small amount of saline and indigo carmine mixture was injected into the submucosa. The mucosa and submucosa of the tumour surface were cut open, and the tumour was exposed. Excavation was performed along the tumour. The tumour was removed using a snare if necessary. The excised samples were recovered and sent to the pathology department for histological diagnosis. We observed whether there was bleeding or perforation on the wound surface and used electrocoagulation for haemostasis in case of bleeding. The wound surface was sutured using tissue clips.

### Clinical outcomes

We analysed the endoscopic complete resection rate, operation time, resection time, and presence of intraoperative perforation, and bleeding. Complete resection was defined as no macroscopic mass remaining after endoscopic tumour resection and negative resection margins according to the histological examination. The operation time was defined as the time from gastroscope insertion to removal from the oral cavity. The resection time was defined as when the transparent cap first contacted the lesion to the completion of wound suturing. Intraoperative perforation was defined as the visualization of intraabdominal organ tissue or omentum. Intraoperative bleeding was defined as macroscopic active blood exudation requiring endoscopic intervention for haemostasis.

### Statistical analysis

All the data were statistically analysed via SPSS28 statistical software. Count data (such as sex, tumour growth pattern, tumour location, and pathological diagnosis) are presented as frequencies, and continuous variables (such as age, tumour size, operation time, and resection time in both groups) are presented as the means ±  SDs. logistic regression was used to assess the relationships between the clinical factors and treatment outcomes (when outcomes were numerical, continuous data were used). Logistic regression (when outcomes were categorical data) was used to test for effect associations among outcomes (dependent variables:intraoperative perforation, and bleeding) and independent variables(independent variables: sex, tumour growth pattern, tumour location, pathological diagnosis,age, tumour size, operation time, and resection time). In the univariate analysis, independent variables were included in the multivariate model with P values <  0.1.All reported p values were two tailed, and p values < 0.05 indicated statistical significance.

## Results

A total of 102 patients were included in this study. The tumours were successfully removed from all the patients. The mean age of the patients was 52.28 ± 11.84 years. Among these patients, 34 patients (33.33%) were male. Endoscopic ultrasound revealed that the mean size of the tumours was 6.96 ± 1.82 mm. Eighty-nine (87.25%) tumours exhibited an intraluminal growth pattern. In total, 79 (77.45%) tumours were in the gastric body, and 23 (22.55%) tumours were in the gastric fundus. The mean operation time was 35.26 ± 23.15 min, and the mean resection time was 27.88 ± 21.77 min. Fifty-five (53.92%) tumours were diagnosed as leiomyoma, and 43 (42.16%) tumours were diagnosed as GIST. There was 1 (0.98%) tumour classified as a fibrous pseudotumour, proliferation of smooth muscle and reactive fibrous pseudotumours. 41 cases (40.20%) had complications due to intraoperative bleeding, all of which had minor bleeding and successful haemostasis under endoscopy. There were 27 (26.47%) concurrent perforations, of which 24(88.89%) were diagnosed as GIST and 3(11.11%) tumours were diagnosed as leiomyoma. All perforations were successfully managed with an endoscopic suture. (Table 1)

**Table 1:** Patient characteristics and treatment outcomes.

Outcomes	Mean±SD (n,%)
No. of patients	102
Age(year)	52.28 ± 11.84
Gender(male)	34(33.33%)
Size of tumour(mm)	6.96 ± 1.82
Location of tumour	
Body	79(77.45%)
Fundus	23(22.55%)
Operation time(min)	35.26 ± 23.15
Resection time(min)	27.88 ± 21.77
Growth pattern	
Intraluminal growth	89(87.25%)
Extraluminal growth	3(2.94%)
Mixed growth	10(9.80%)
Histology diagnosis	
Leiomyoma	55(53.92%)
GIST	43(42.16%)
Calcified fibrous pseudotumour	1(0.98%)
Proliferation of smooth muscle	1(0.98%)
Reactive fibrous pseudotumours	1(0.98%)
Interstitial collagen fibrous hyperplasia	1(0.98%)
Intraoperative adverse events	
Bleeding	41(40.20%)
Perforation	27(26.47%)
GIST	24(88.89%)
Leiomyoma	3(11.11%)

*GIST* gastrointestinal stromal tumour.

According to the multivariate regression analysis, a pathologic diagnosis was a risk factor for perforation. When the pathological diagnosis is GIST, the risk of perforation increases (PE = 18.632, 95% CI 4.571 ~ 75.941; p < 0.001). Gender,age,tumor size, growth pattern, location, and resection time were not found to be risk factors for perforation.The results of the multivariate regression analysis showed that gender,age,tumour size, growth pattern, location, pathologic diagnosis, and resection time were not risk factors for bleeding (Table 2).

**Table 2:** Multivariate regression analysis for perforation and bleeding.

Characteristics	Perforation, PE 95% CI	Bleeding, PE 95% CI
Gender	1.724(0.503 ~ 5.914)	2.197(0.839 ~ 5.752)
Age	1.006(0.959 ~ 1.057)	0.980(0.941 ~ 1.021)
Size in EUS	1.071(0.748 ~ 1.535)	1.022(0.776 ~ 1.348)
Location of tumour	1.842(0.510 ~ 6.648)	0.336(0.104 ~ 1.086)
Growth pattern	0.400(0.060 ~ 2.678)	0.443(0.082 ~ 2.394)
Resection time	1.017(0.991 ~ 1.045)	1.023(1.000 ~ 1.047)
Histology diagnosis	18.632(4.571 ~ 75.941)*	0.373(0.133 ~ 1.042)

*PE* parameter estimate, *CI* confidence interval.

*p < 0.05.

## Discussion

Endoscopic resection methods for gastric muscularis propria tumours include endoscopic submucosal dissection (ESD), ESE, submucosal tunnel endoscopic resection (STER), and endoscopic full-thickness resection (EFTR) [5–8]. Selection of treatment method depends on the experience of the operators, the medical level of the hospital, and the size and location of the tumour. ESE is a relatively mature and widely used endoscopic resection method [9]. The reduction and prevention of intraoperative adverse events in patients with ESE is particularly important. Uncontrolled bleeding or perforation is also a considerable technical and psychological challenge for experienced endoscopists.

Common intraoperative adverse events of ESE include perforation and bleeding. Jinshun Zhang et al. [10] performed ESE resection of 69 gastric GISTs < 30 mm, which showed that 5 patients experienced intraoperative bleeding and 23 patients experienced perforation, with a perforation rate of 18% when the mass protruded into the gastric cavity and 73.68% when the mass protruded outside of the gastric cavity. Liping Ye et al. [11] performed ESE resections on 116 tumours originating from the gastric muscularis propria, including 20 cases of intraoperative perforation, 9 cases of intraoperative bleeding, and 3 cases that affected endoscopic operation vision, thus transferring them to surgical surgery. In one patient, the mass fell into the abdominal cavity, and was removed laparoscopically. Our study presented the same results, which revealed a high incidence of perforation(26.47%) and bleeding(40.20%) during ESE. These results indicate that although ESE is a widely used endoscopic resection method, the incidence of intraoperative perforation and bleeding is high, and surgery is needed when bleeding is severe, which can cause great damage to patients and increase medical burden.

GISTs are classified into four types according to the growth pattern of the tumour by Kim H H [12]: type Ⅰ GIST refers to a tumour with a very narrow connection with the proper muscle layer (PM), protruding into the gastric lumen, similar to polyps; type II tumours have a wider connection with the PM and protrude into the lumen at an obtuse angle; type III lesions are located in the middle of the gastric wall; and type IV tumours protrude mainly into the serosal side of the gastric wall. The results of the study revealed that the tumour growth pattern may result in perforation. As shown by the findings of Kim et al. [12], all perforations occurred in patients with GISTs and schwannomas, which may have been due to the poor tumour capsule and tight adhesions. The location of gastric GISTs is another factor that may cause perforation. Jeong ID et al. [13] reported that the incidence of perforation at the fundus was greater than that at other locations. These results suggest that the tumour growth pattern, location, and pathological diagnosis are risk factors for perforation.

For gastric muscularis propria tumours less than 10 mm, there are no relevant reports on the risk factors leading to adverse events during ESE resection. Our results revealed that intraoperative perforation occurred in both leiomyomas and GISTs, but GIST accounted for 88.89% of cases. Thus, GIST was the main risk factor for perforation, whereas gender,age,tumour size, growth pattern, location, and operation time were not risk factors for perforation.This may be because GIST closely adheres closely to the lamina propria and does not easily separate propria and tumour during resection, which increases the risk of perforation.The leiomyoma has a capsule that easily separates the tumour and the muscularis propria during resection, which reduces the damage to the muscularis propria and avoids perforation.This result suggests that if the tumour is considered GIST before surgery, we should be prepared for perforation during the procedure.The operator needs to prepare antibiotics, abdominal puncture and exhaust tools, suture tools in advance.So as to reduce or even avoid the occurrence of pneumata, abdominal infection and other serious adverse events caused by perforation.

The findings presented herein are not entirely consistent with previous findings, and may be due to the inclusion of tumors that were only less than 10 mm in diameter For small muscularis propria tumours, there are large deviations in the evaluation of the tumour growth pattern by endoscopic ultrasonography. Because the tumour is small enough, even if it grows in the thin fundus of the stomach wall, because the lesion does not fully accumulate in the full layer of the propria muscle, part of the muscularis propria component can still be retained during the process of ESE, thus avoiding the need for full-thickness resection.

The results of this study revealed that 41 patients (40.20%) experienced intraoperative bleeding. All patients who experienced minimal bleeding were successfully managed via endoscopic treatment. The results of multivariate regression analysis revealed that the gender,age,tumour size, location, growth pattern, resection time, and pathologic diagnosis were not risk factors for bleeding. This finding suggests that bleeding is very common when resection of the gastric muscularis propria tumour is less than 10 mm, and operators need to overcome panic because this type of bleeding is mostly manageable.

Our study has several limitations. First, the number of cases was limited because this was a retrospective study,which may impact the results. Second, in this study, three endoscopists performed endoscopic resection. We cannot avoid differences in the ability of our staff in terms of endoscopic treatment, but all endoscopists had more than 5 years of endoscopic experience, and all completed more than 200 cases of ESE. Third, only gastric muscularis propria tumours less than 10 mm in size were included in this study, and whether the conclusions are suitable for larger tumours need further study.

In conclusion, bleeding and perforation during ESE are common complications. For gastric muscularis propria tumours less than 10 mm, the histological type should be strictly evaluated before ESE. For tumours with highly suspected GIST, perforation should be prepared in advance for ESE to help reduce serious adverse events caused by perforation (including abdominal infection, tumours falling into the abdominal cavity, etc.).

## Supporting information

S1 Table Patient characteristics and treatment outcomes. (XLSX)

S2 Table Multivariate regression analysis for perforation and bleeding. (XLSX)

S1 File Dates. (XLSX)
